# Situational analysis of diabetic retinopathy treatment Services in Ghana

**DOI:** 10.1186/s12913-021-06608-9

**Published:** 2021-06-17

**Authors:** Agatha Mensah-Debrah, Kwesi Nyan Amissah Arthur, David Ben Kumah, Kwadwo Owusu Akuffo, Isaiah Osei Duah, Covadonga Bascaran

**Affiliations:** 1 National Eye Care Unit, Ghana Health Service, Accra, Ghana; 2 International Centre for Eye Health, London School of Hygiene and Tropical Medicine, London, UK; 3 Ophthalmology Unit, Department of Surgery, Korle Bu Teaching Hospital, College of Health Sciences, School of Medicine and Dentistry, University of Ghana, Accra, Ghana; 4 Department of Optometry and Visual Science, Kwame Nkrumah University of Science and Technology, Kumasi, Ghana

**Keywords:** Cost-utility analysis, Anti-VEGF, Vitreoretinal surgery, Retinal laser, Diabetic retinopathy treatment, Ghana, Disparities in health care, Barrier to diabetic treatment

## Abstract

**Background:**

Although the equitable distribution of diabetic retinopathy (DR) services across Ghana remains paramount, there is currently a poor understanding of nationwide DR treatment services. This study aims to conduct a situation analysis of DR treatment services in Ghana and provide evidence on the breadth, coverage, workload, and gaps in service delivery for DR treatment.

**Methods:**

A cross-sectional study was designed to identify health facilities which treat DR in Ghana from June 2018 to August 2018. Data were obtained from the facilities using a semi-structured questionnaire which included questions identifying human resources involved in DR treatment, location of health facilities with laser, vitreoretinal surgery and Anti–vascular endothelial growth factor therapy (Anti-VEGF) for DR treatment, service utilisation and workload at these facilities, and the average price of DR treatment in these facilities.

**Results:**

Fourteen facilities offer DR treatment in Ghana; four in the public sector, seven in the private sector and three in the Christian Health Association of Ghana (CHAG) centres. There was a huge disparity in the distribution of facilities offering DR services, the eye care cadre, workload, and DR treatment service (retinal laser, Anti-VEGF, and vitreoretinal surgery). The retinal laser treatment price was independent of all variables (facility type, settings, regions, and National Health Insurance Scheme coverage). However, settings (*p* = 0.028) and geographical regions (*p* = 0.010) were significantly associated with anti-VEGF treatment price per eye.

**Conclusion:**

Our results suggest a disproportionate distribution of DR services in Ghana. Hence, there should be a strategic development and implementation of an eye care plan to ensure the widespread provision of DR services to the disadvantaged population as we aim towards a disadvantaged population as we aim towards a universal health coverage.

**Supplementary Information:**

The online version contains supplementary material available at 10.1186/s12913-021-06608-9.

## Background

Globally, the number of people living with diabetes is estimated to be 451 million, and this figure is projected to increase to 693 million by the year 2045 if no serious and committed action is taken [1]. In Sub-Saharan Africa and other developing countries, diabetes prevalence is known to have risen more quickly than predicted, from 12.1 million in 2010 to 15.5 million in 2017 [2, 3]. Rapid urbanisation, globalisation and unhealthy lifestyle are widely acknowledged to have contributed to the growing epidemic of diabetes worldwide and in Africa [4]. A case in point is a developing country such as Ghana experiencing a rapid increase in diabetes prevalence from 0.2% in 1964, to 1.9% and 6.46% in 2010 and 2018, respectively [2, 5].

One of the major complications of diabetes is the development of diabetic retinopathy (DR) [6]. Previous studies have shown that approximately one-third of people living with diabetes will develop DR and a third of those with DR will develop vision-threatening diabetic retinopathy (VTDR) [6]. Moreover, the number of people with DR is projected to increase from 126.6 million in 2010 to 191.0 million by 2030, whilst global estimates show the number with VTDR will increase from 37.3 million to 56.3 million if immediate action is not taken [7]. DR is the leading cause of vision loss in the working-age population worldwide [6, 8]. Few studies have been done on DR in Ghana, and all were centred on specific health facilities in the country using different age cut-offs. With the recent increase of diabetes in Ghana, retinopathies are set to rise, especially with life expectancy improvement. Therefore, it is imperative to know what services are in place to treat DR and where the gaps are to enable the planning of a more systematic DR screening service nationwide.

Though services for diabetic management have improved in recent years [9], screening and management for DR are not fully incorporated into the national diabetes program [9, 10]. Integrating eye care services into the general health system helps strengthen the system for effective planning and service delivery [11, 12].

This study aims to conduct a situation analysis of DR treatment services in Ghana and provide evidence on the breadth, coverage, workload, and gaps in service delivery for DR treatment. Identifying these gaps will inform decisions and policies on where resources should be allocated. A robust treatment service on offer is essential when considering a more comprehensive population-based screening in the foreseeable future.

## Materials and methods

### Study design

The study employed a cross-sectional study design. All health facilities (e.g., public, private, faith-based or Non-Governmental Organisation (NGO)-based health facilities) identified to offer treatment services for DR in Ghana were included in the study. Since this study’s purpose was to conduct a comprehensive situational analysis countrywide, all health facilities identified to offer treatment for DR in Ghana were included.

### Study setting

**Fig. 1:**
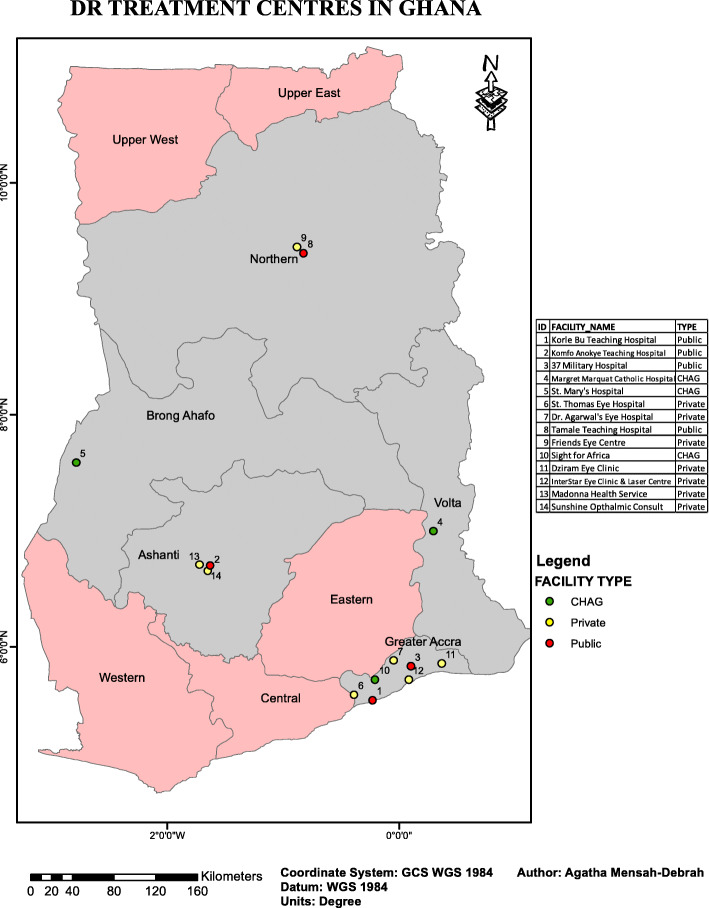
Location and type of facility delivering treatment for Diabetic Retinopathy in Ghana

Ghana is a lower middle-income country in West Africa located on the coast of the Gulf of Guinea with Accra as the capital. With a population of 24,658,823 and a growth rate of 2.5% according to the 2010 census [13], Ghana has one of the fastest growing populations in Africa. In 2017, Ghana’s population increased to 28,833,629 [14]. The country has a GDP per capita of 1513.46 USD and total expenditure on health is 3.6% [15]. Life expectancy is estimated at 61.7 years and the country has an under five mortality rate of 78 per 1000 live births [15]. The average poverty rate in Ghana is far higher in rural areas compared to urban centres in Ghana (37.9% for rural and 10.6% for urban). In addition, the average poverty reduction is far faster in urban areas than in rural areas (poverty rate is 4 times higher in rural areas than in urban areas) [16]. The Upper East and Upper West Regions in Ghana on average have a third of its population living under the poverty line [16]. Figure 1 is a map of Ghana showing the distribution of DR services across the ten administrative regions (please note that the administrative regions were increased to sixteen after the study was completed).

### Eligibility criteria

All hospitals or clinics identified by regional ophthalmologists to offer some form of treatment for VTDR (laser, Anti-VEGF, VR surgery) in Ghana were included. Hospitals or clinics could be Government-based, faith-based, Quasi, private or NGO-based.

Christian Health Association of Ghana (CHAG) is a form of public private partnership in healthcare delivery. CHAG is a recognised agency (i.e., faith-based) of the Ministry of Health made up of a network of 344 health facilities and health training institutions owned by 33 different Christian Church Denominations. CHAG provides health care to the most vulnerable and underprivileged population groups in all regions of Ghana, particularly in the most remote areas.

Quasi facilities are facilities setup or supported by government, but managed privately.

Hospitals or Eye clinics in Ghana that did not offer any form of treatment for VTDR before 31st December 2017 were excluded from the study.

### Participant recruitment

**Fig. 2:**
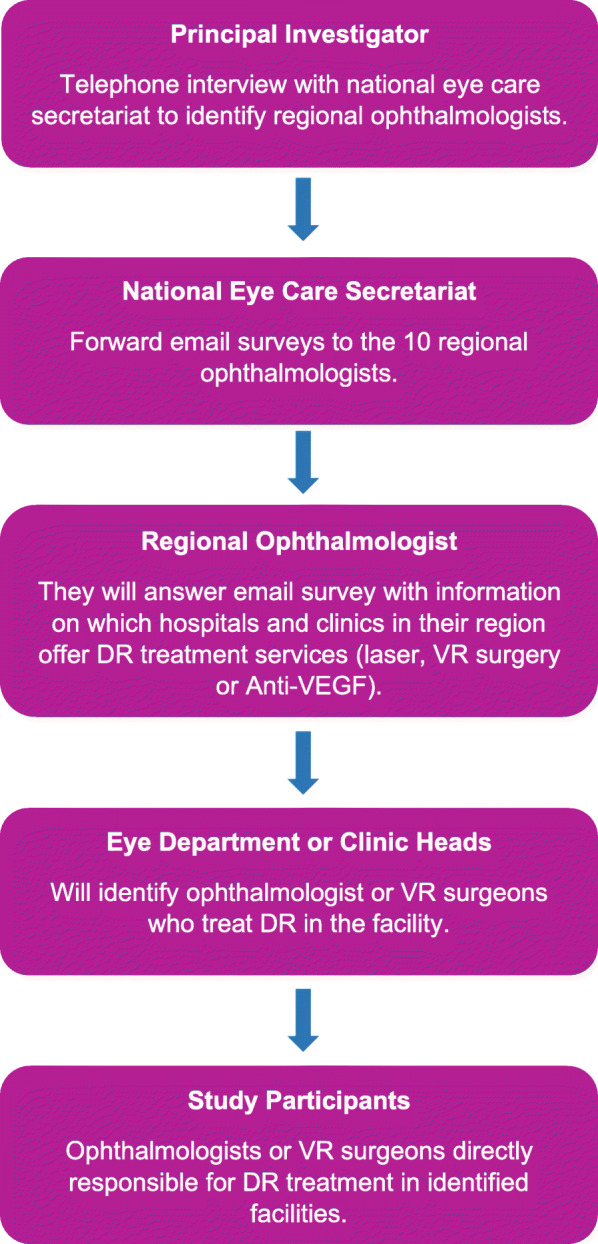
Identification of study participants

Study participants were ophthalmologists or vitreoretinal surgeons treating DR in identified facilities. To find such facilities, a survey questionnaire was sent to all regional ophthalmologists through an email forwarded by the National Eye Care Secretariat to identify study participants (Fig. 2). Regional ophthalmologists coordinate all regional eye care activities and have access to the database of regional eye care providers and their contacts. Regional ophthalmologists answered questions pertaining to which hospitals or clinics offer treatment for VTDR within their region since most treatments are done by ophthalmologists or Vitreoretinal (VR) surgeons. Hospitals or Clinic heads were then contacted to identify the person or persons responsible for DR treatment in their facility.

### Data collection

Semi-structured questionnaires were administered by the Principal Investigator (PI) to study participants to identify and assess DR treatment services in Ghana. The semi-structured questionnaire had questions aimed at identifying human resources involved in DR treatment, location of health facilities which have retinal laser, VR surgery and Anti-VEGF for DR treatment, service utilisation and workload at these facilities, and the average price of DR treatment in these facilities. DR registers and records were examined to identify the number of retinal lasers, Anti-VEGF and VR surgeries performed in 2017. A checklist was also used to identify equipment used in DR treatment. Data collection covered a period from 11th June 2018 to 13th August 2018.

### Quality control

Three Ophthalmologists in Africa, working in facilities with retinal laser took part in their work facilities in Africa took part in the pretesting of questionnaires. The questionnaires were edited as a result of the feedback received from the pretest. Questionnaires were written in English, which is the official language of Ghana. The principal investigator administered questionnaires and checklists personally. Data entered was double-checked to minimise errors.

### Ethical approval

Ethical approval was obtained from the institutional review committees at the London School of Hygiene and Tropical Medicine (Reference Number: 15308) and the institutional review boards of three major hospitals in Ghana; namely Korle Bu Teaching Hospital (Reference Number: KBTH-IRB/00060/2018), Komfo Anokye Teaching Hospital (Reference Number: CHRPE/AP/328/18) and 37 Military Hospital (Reference Number: 37MH-IRB IPN/218/2018). Informed consent and permission were obtained from facility heads and study participants before interviews took place. Participant names were not included in questionnaires and facility names were excluded in reports. Facility names were replaced with codes to ensure confidentiality.

### Data analysis

Data analysis was performed using Statistical Product and Service Solution (IBM Corporation IBM® SPSS® Statistics for Windows, Version 25.0 Armonk, NY) compatible with Windows 10. Multiple data cleaning was performed as part of data quality checks. The analytic sample comprised the health facilities treating DR. Descriptive statistics were used to assess the frequencies and proportions of demographic variables (facility type, settings, regions, and other DR service parameters). The association between demographic variables and the price of DR treatments were investigated using a one-way analysis of variance (ANOVA). Statistical significance was set at *p* < 0.05.

## Results

Fourteen eye care facilities were identified to fulfil the inclusion criteria and all of them agreed to participate in the study (see Fig. 1 and Table 1). All facilities participated in the study (100% response rate). Seven facilities were private, four were public and three were faith-based, part of the CHAG.

**Table 1:** List of Eligible Eye Care Facilities

N	Facility Name	Region	Type of facility
1	Korle Bu Teaching Hospital	Greater Accra	Public
2	Komfo Anokye Teaching Hospital	Ashanti	Public
3	37 Military Hospital	Greater Accra	Public
4	Margret Marquart Catholic Hospital	Volta	CHAG
5	St. Mary’s Hospital	Brong-Ahafo	CHAG
6	St. Thomas Eye Hospital	Greater Accra	Private
7	Dr. Agarwal’s Eye Hospital	Greater Accra	Private
8	Tamale Teaching Hospital	Northern	Public
9	Friends Eye Centre	Northern	Private
10	Sight for Africa	Greater Accra	CHAG
11	Dziram Eye Clinic	Greater Accra	Private
12	Interstar Eye Clinic and Laser Centre	Greater Accra	Private
13	Madonna Health Services	Ashanti	Private
14	Sunshine Ophthalmic Consult	Ashanti	Private

### Diabetic retinopathy treatment facilities

Table 2 presents the demographic profile of the fourteen facilities offering DR treatment services in Ghana. The majority were private facilities (50.0%) and were located in urban settings (78.6%). Most of the facilities were concentrated in the Greater Accra (50.0%) and Ashanti (21.4%) and with a fewer in the Brong Ahafo (7.1%) and Volta Regions (7.1%).

**Table 2:** Description of the sample

Variable	n	%
**Facility type**		
Public	4	28.6
CHAG	3	21.4
Private	7	50.0
**Setting**		
Rural	3	21.4
Urban	11	78.6
**Regions with diabetic retinopathy treatment services**		
Greater Accra Region	7	50.0
Ashanti Region	3	21.4
Northern Region	2	14.3
Brong Ahafo Region	1	7.1
Volta Region	1	7.1
**Guidelines for diabetic retinopathy**		
ICO guidelines	6	33.3
Clinical expertise	12	66.7
**Routine diabetic examination**		
No	7	50.0
Yes	7	50.0
**Referral pathway**		
One way	5	35.7
Reciprocal	9	64.3
**Diabetic retinopathy treatments**		
Retinal Laser	10	35.7
Anti-VEGF	12	42.9
Vitreoretinal surgery	6	21.4
**Health professionals treating diabetic retinopathy**		
Vitreoretinal surgeon	11	28.2
Ophthalmologists	19	48.7
Optometrists	1	2.6
Ophthalmic nurses	8	20.5
**Continuing medical education**		
Formal training by a regulated body	7	25.9
Regular informal updates	5	18.5
Workshops	9	33.3
Updates on guidelines	6	22.2
**Records on diabetic retinopathy treatments**		
No	3	21.4
Yes	11	78.6
**Indicators that are monitored**		
Type of treatment	11	26.8
Eye treated	11	26.8
Number of times eye treated	9	22.0
Visual acuity after treatment	10	24.4
**NHIS coverage**		
No	11	78.6
Yes	3	21.4
**NHIS services on diabetic retinopathy**		
Diabetes care	3	33.3
DR screening	3	33.3
Laser photocoagulation	1	11.1
Anti-VEGF	1	11.1
Vitreoretinal surgery	1	11.1

*n* frequency of facilities, *%* percentage frequency of facilities, *CHAG* Christian Health Association of Ghana, *Anti-VEGF* Anti-vascular endothelial growth factor therapy, *NHIS* National Health Insurance Scheme

Most of the facilities treated DR based on the clinical expertise defined as the proficiency and judgement made by individual clinicians acquired through experience and practice intended to optimize or improve patient care (66.7%). A greater proportion of the facilities (64.3%) reported using a reciprocal referral pathway; thus, physicians at the same or higher level of health care system refer to each other and vice versa with the aim of getting feedback from referral facilities. With respect to DR treatments, the following were recorded for the procedures: Anti-VEGF (42.9%); retinal laser photocoagulation (35.7%) and vitreoretinal surgery (21.4%). The majority (51.9%) of the facilities indicated that an ophthalmologist treated DR, and most (33.3%) received continuing medical education through workshops. A preponderance (78.6%) of the facilities had DR treatment records; however, many (78.6%) had no NHIS coverage (Table 2).

### Human resource treating diabetic retinopathy in Ghana

Non-surgical DR treatment in Ghana is delivered by 19 General Ophthalmologists, 11 vitreoretinal surgeons, eight ophthalmic nurses and one optometrist. The distribution of these eye care professionals across setting, facility types and regions in Ghana is shown in Table 3. All 11 vitreoretinal surgeons (100%) and one optometrist were only practising in private urban facilities. The majority of ophthalmologists and ophthalmic nurses were practising in an urban setting (78.9% versus 62.5%, respectively).

**Table 3:** Distribution of personnel, workload, and diabetic retinopathy treatment services in Ghana

Variable	Setting		Facility type			Regions				
	Rural	Urban	Public	CHAG	Private	GAR	AR	NR	BAR	VR
	n (%)	n (%)	n (%)	n (%)	n (%)	n (%)	n (%)	n (%)	n (%)	n (%)
**Health professionals treating DR**										
Vitreoretinal surgeon	0 (0.0)	11 (100.0)	4 (36.36)	1 (9.09)	6 (54.55)	10 (90.9)	1 (9.1)	0 (0.0)	0 (0.0)	0 (0.0)
Ophthalmologist	4 (21.1)	15 (78.9)	6 (31.6)	3 (15.8)	10 (52.6)	10 (52.63)	4 (21.05)	3 (15.79)	1 (5.26)	1 (5.26)
Optometrist	0 (0.0)	1 (100.0)	0 (0.0)	0 (0.0)	1 (100)	1 (100.0)	0 (0.0)	0 (0.0)	0 (0.0)	0 (0.0)
Ophthalmic nurse	3 (37.5)	5 (62.5)	0 (0.0)	3 (37.5)	5 (62.5)	5 (62.5)	0 (0.0)	0 (0.0)	0 (0.0)	3 (37.5)
**DRT**										
Retinal laser photocoagulation	3 (30.0)	7 (70.0)	2 (20.0)	3 (30.0)	5 (50.0)	6 (60.0)	1 (10.0)	1 (10.0)	1 (10.0)	1 (10.0)
Anti-VEGF	1 (9.1)	11 (90.9)	4 (33.33)	1 (8.33)	7 (58.33)	7 (58.3)	3 (25.0)	2 (16.7)	0 (0.0)	0 (0.0)
Vitreoretinal surgery	0 (0.0)	6 (100.0)	2 (33.3)	0 (0.0)	4 (66.7)	5 (83.3)	1 (16.7)	0 (0.0)	0 (0.0)	0 (0.0)
**Guidelines for DRT**			3 (33.3)							
ICO guidelines	1 (16.7)	5 (83.3)	2 (33.3)	1 (16.7)	3 (50.0)	2 (33.3)	2 (33.3)	1 (16.7)	1 (16.7)	0 (0.0)
Clinical expertise	3 (25.0)	9 (75.0)	4 (33.3)	3 (25.0)	5 (41.7)	5 (41.7)	3 (25.0)	2 (16.7)	1 (8.3)	1 (8.3)
**Routine diabetic examination**	2 (28.6)	5 (71.4)	3 (42.86)	2 (28.57)	2 (28.57)	4 (57.14)	0 (0.0)	1 (14.29)	1 (14.29)	1 (14.3)
**Referral pathway**										
One-way	0 (0.0)	5 (100.0)	1 (20.0)	1 (20.0)	3 (60.0)	4 (80.0)	1 (20.00)	0 (0.0)	0 (0.0)	0 (0.0)
Reciprocal	3 (33.3)	6 (66.7)	3 (33.33)	2 (22.22)	4 (44.44)	3 (33.33)	2 (22.22)	2 (22.22)	1 (11.11)	1 (11.1)
**Records availability**	3 (27.3)	8 (72.7)	2 (18.18)	3 (27.27)	6 (54.55)	5 (45.45)	2 (18.18)	2 (18.18)	1 (9.09)	1 (9.09)
**Number of people treated**										
Retinal laser	33 (1.9)	1669 (98.1)	293 (17.22)	117 (6.87)	1292 (75.91)	1461 (85.84)	208 (12.22)	5 (0.29)	10 (0.59)	18 (1.1)
Anti-VEGF	15 (0.8)	1953 (99.2)	268 (13.62)	124 (6.30)	1576 (80.08)	1716 (87.20)	229 (11.64)	23 (1.17)	0 (0.0)	0 (0.0)
Vitreoretinal surgery	0 (0.0)	407 (100.0)	22 (5.41)	21 (5.16)	364 (89.43)	407 (100.0)	0 (0.0)	0 (0.0)	0 (0.0)	0 (0.0)
**Number of eyes treated**										
Retinal laser photocoagulation	37 (1.3)	2910 (98.7)	406 (13.78)	117 (3.97)	2424 (82.25)	2589 (87.85)	321 (10.89)	9 (0.31)	10 (0.34)	18 (0.6)
Anti-VEGF	21 (0.6)	3435 (99.4)	338 (9.78)	124 (3.59)	2994 (86.63)	3103 (89.79)	318 (9.20)	35 (1.01)	0 (0.0)	0 (0.0)
Vitreoretinal surgery	0 (0.0)	533 (100.0)	22 (4.13)	21 (3.94)	490 (91.93)	533 (100.0)	0 (0.0)	0 (0.0)	0 (0.0)	0 (0.0)
**Checklist for DRT services**										
Screening protocols for DM	2 (28.6)	5 (71.4)	4 (57.14)	1 (14.29)	2 (28.57)	3 (42.86)	1 (14.29)	2 (28.57)	1 (14.29)	0 (0.00
Screening protocols for DR	1 (11.1)	8 (88.9)	4 (44.44)	1 (11.11)	4 (44.44)	6 (66.67)	1 (11.11)	2 (22.22)	0 (0.0)	0 (0.0)
Grading protocols for DR	1 (11.1)	8 (88.9)	4 (44.44)	1 (11.11)	4 (44.44)	6 (66.67)	1 (11.11)	2 (22.22)	0 (0.0)	0 (0.0)
Treatment protocols for DR	1 (11.1)	8 (88.9)	4 (44.44)	1 (11.11)	4 (44.44)	6 (66.67)	1 (11.11)	2 (22.22)	0 (0.0)	0 (0.0)
DR treatment registry/records	2 (18.2)	9 (81.8)	3 (27.27)	2 (18.18)	6 (54.55)	7 (63.64)	1 (9.09)	2 (18.18)	1 (9.09)	0 (0.0)
Anti-VEGF drugs	1 (8.3)	11 (91.7)	4 (33.33)	1 (8.33)	7 (58.33)	7 (58.33)	3 (25.00)	2 (16.67)	0 (0.0)	0 (0.0)
27 needle and 1 ml syringe	2 (15.4)	11 (84.6)	4 (30.77)	2 (15.38)	7 (53.85)	7 (53.85)	3 (23.08)	2 (15.38)	0 (0.0)	1 (7.69)
Operating microscope	3 (21.4)	11 (78.6)	4 (28.57)	3 (21.43)	7 (50.00)	7 (50.00)	3 (21.43)	2 (14.29)	1 (7.14)	1 (7.14)
Retinal Laser machine	2 (20.0)	8 (80.0)	3 (30.00)	3 (30.00)	4 (40.00)	7 (70.00)	1 (10.0)	0 (0.0)	1 (10.00)	1 (10.0)
Vitrectomy machine	1 (12.5)	7 (87.5)	2 (25.00)	2 (25.00)	4 (50.00)	6 (75.00)	1 (12.5)	0 (0.0)	0 (0.0)	1 (12.5)
Fundus/retinal camera	2 (22.2)	7 (77.8)	2 (22.22)	2 (22.22)	5 (55.56)	5 (55.56)	2 (22.22)	1 (11.11)	1 (11.11)	0 (0.0)
OCT machine	0 (0.0)	7 (100.0)	3 (42.86)	1 (14.29)	3 (42.86)	6 (85.7)	1 (14.3)	0 (0.0)	0 (0.0)	0 (0.0)
Pan retinal photocoagulation lens	1 (10.0)	9 (90.0)	3 (30.0)	2 (20.0)	5 (50.0)	7 (70.00)	2 (20.00)	0 (0.0)	0 (0.0)	1 (10.0)
FFA/ICG angiography	1 (16.7)	5 (83.3)	2 (33.3)	1 (16.7)	3 (50.0)	4 (66.67)	1 (16.67)	0 (0.0)	0 (0.0)	1 (16.7)
Slit lamp biomicroscope	3 (21.4)	11 (78.6)	4 (28.6)	3 (21.4)	7 (50.0)	7 (50.0)	3 (21.43)	2 (14.29)	1 (7.14)	1 (7.14)
Direct ophthalmoscope	3 (21.4)	11 (78.6)	4 (28.6)	3 (21.4)	7 (50.0)	7 (50.00)	3 (21.43)	2 (14.29)	1 (7.14)	1 (7.14)
Indirect ophthalmoscope	3 (21.4)	11 (78.6)	4 (28.6)	3 (21.4)	7 (50.0)	7 (50.00)	3 (21.43)	2 (14.29)	1 (7.14)	1 (7.14)
90 D lens	3 (23.08)	10 (76.92)	4 (30.77)	3 (23.08)	6 (46.15)	7 (53.85)	2 (15.38)	2 (15.38)	1 (7.69)	1 (7.69)
78 D lens	2 (15.4)	11 (84.6)	4 (30.77)	2 (15.38)	7 (53.85)	7 (53.85)	3 (23.08)	2 (15.38)	1 (7.69)	0 (0.0)
20 D lens	3 (21.4)	11 (78.6)	4 (28.57)	3 (21.43)	7 (50.0)	7 (50.0)	3 (21.43)	2 (14.29)	1 (7.14)	1 (7.14)
Three-mirror contact lens	2 (16.7)	10 (83.3)	4 (33.3)	2 (16.7)	6 (50.0)	7 (58.33)	2 (16.67)	2 (16.67)	0 (0.0)	1 (8.33)
Visual acuity chart	3 (21.4)	11 (78.6)	4 (28.6)	3 (21.4)	7 (50.0)	7 (50.0)	3 (21.43)	2 (14.29)	1 (7.14)	1 (7.14)

*n* frequency, *%* percentage frequency, *CHAG* Christian Health Association of Ghana, *GAR* Greater Accra Region, *AR* Ashanti Region, *NR* Northern Region, *BAR* Brong Ahafo Region, *VR* Volta Region, *DRT* Diabetic Retinopathy Treatments, *Anti-VEGF* Anti-vascular endothelial growth factor therapy, *DM* Diabetes mellitus, *DR* Diabetic retinopathy, *OCT* Optical Coherence Tomography, *FFA* Fluorescein Angiography, *ICG* Indocyanine Green, *D* Dioptres; Workload is defined as the total number of eyes treated for DR using retinal lasers, Anti-VEGF or by VR surgery

In terms of distribution across the regions of Ghana, eye care professionals were found in only five regions of Ghana; namely Greater-Accra, Ashanti, Northern and Brong Ahafo, Volta), with ophthalmologists having a representation in all four regions.

The distribution of the total workload across setting, facility types and regions in Ghana is shown in Table 3. Workload is defined as the total number of eyes treated for DR using retinal lasers, Anti-VEGF, or VR surgery. The total number of people treated is the total number of persons who visited the health facilities for DR treatment (the number of treatments received by a person is counted as one).

### Equipment for diabetic retinopathy treatment in Ghana

There are variations in the geographical location and distribution of equipment used in DR treatment (Table 3). A total of nine retinal cameras, ten retinal laser machines and seven OCT machines were enumerated in the study. Interestingly, most of the retinal lasers (70%) are concentrated in the Greater Accra region, with only one retinal laser located in the Ashanti, Brong Ahafo and Volta Regions of Ghana. Similarly, most retinal cameras are concentrated in the Greater Accra region (55.6%). All OCT machines were found in urban settings (100%). Thus, there is a gap in equipment availability, especially in the extreme north and south-western part of Ghana.

### Utilisation of diabetic retinopathy treatment Services in Ghana

The level of utilization of DR treatment Services utilisation (in terms of the number of eyes treated) in Ghana is also shown in Table 3. The majority of retinal lasers to treat DR were performed in private facilities (82.3%) and urban settings (98.7%). Similarly, Anti-VEGF treatment for DR was done in the private sector facilities (86.6%) and urban settings (99.4%). Of note, all vitreoretinal surgeries were performed in urban settings and the Greater Accra region.

### Price of diabetic retinopathy treatment in Ghana

The price of recommended DR treatments (anti-VEGF injection and retinal laser photocoagulation) varied across different settings, facility types and regions in Ghana (Table 4). No factor was significantly associated (*p* > 0.05, for all) with price per retinal laser treatment in our analyses. However, geographical settings (F-statistic F (1,12) = 6.22, *p* = 0.028) and Regions (F-statistic F (4,9) = 6.38, *p* = 0.010) significantly influenced price per eye of Anti-VEGF treatments. Post Hoc test for the Regions Variable was not statistically feasible because two subgroups had fewer than two cases. The price per eye of Anti-VEGF treatment on average was significantly higher in the urban settings (500GHS) compared to the rural setting (133.3GHS). The average price in the Greater Accra region was more than two times higher compared to that in the Ashanti region of Ghana.

**Table 4:** Comparison of Cost of Diabetic Retinopathy Treatment to Annual Average Incomes in Ghana

	**Price per eye Retinal laser GH¢)**						**Mean laser price as % of**	**Mean laser price as % of**
	n	Mean	Std. Deviation	Minimum	Maximum	p - value	**Mean household income (GH¢)**	**mean annual per capita income (GH¢)**
**Facility type**								
Public	4	262.50	205.65	0.00	450.00	0.499		
CHAG	3	200.00	100.00	100.00	300.00			
Private	7	428.57	380.63	0.00	1000.00			
**Setting**								
Rural	3	183.33	76.38	100.00	250.00	0.349		
Urban	11	372.73	325.09	0.00	1000.00			
**Regions**								
GAR	7	528.57	279.67	200.00	1000.00	0.175	0.82	2.45
AR	3	133.33	230.94	0.00	400.00		0.18	0.24
NR	2	125.00	176.78	0.00	250.00		0.55	2.17
BAR	1	200.00	.	200.00	200.00		0.65	1.59
VR	1	100.00	.	100.00	100.00		0.32	1.35
NHIS Coverage								
No	11	331.82	327.32	0.00	1000.00	0.994		
Yes	3	333.33	202.07	100.00	450.00			
	**Price per eye Anti-VEGF GH¢)**						**Mean anti-VEGF price as % of mean household income (GH¢)**	**Mean anti-VEGF price as % of mean annual per capita income (GH¢)**
**Facility type**								
Public	4	487.50	232.29	250.00	800.00	0.181		
CHAG	3	166.67	288.68	0.00	500.00			
Private	7	492.86	242.26	200.00	950.00			
**Setting**								
Rural	3	133.33	230.94	0.00	400.00	0.028		
Urban	11	500.00	224.72	200.00	950.00			
**Regions**								
GAR	7	607.14	196.70	400.00	950.00	0.010	0.94	2.81
AR	3	250.00	50.00	200.00	300.00		0.34	0.44
NR	2	450.00	70.71	400.00	500.00		1.96	7.83
BAR	1	0.00	.	0.00	0.00		0.00	0.00
VR	1	0.00	.	0.00	0.00		0.00	0.00
**NHIS Coverage**								
No	11	413.64	216.90	0.00	800.00	0.181		
Yes	3	450.00	476.97	0.00	950.00			

*n* number of facilities, *Anti-VEGF* Anti-vascular endothelial growth factor therapy, *CHAG* Christian Health Association of Ghana, *NHIS* National Health Insurance Scheme, *GAR* Greater Accra Region, *AR* Ashanti Region, *NR* Northern Region, *BAR* Brong Ahafo Region, *VR* Volta Region, *Std. Deviation* standard deviation; Statistical test employed one-way Analysis of variance (ANOVA) and significance set at *p* < 0.05. Mean annual household income (GH¢) and Mean annual per capita income (GH¢) is based on the Ghana Living Standards Survey Round 7 (GLSS-7) Report. Report [17]

### National assessment of diabetic retinopathy treatment services

The Ghana National Eye Care Unit completed a structured questionnaire aimed at understanding DR treatment at the national level. From the completed questionnaire, Ghana has a national health plan (which needs an update) and a national diabetes health plan covering primary prevention of blindness; community awareness and patient education; clinical care, services and supplies; and some complications but not including visual impairment. Ghana has a national prevention of blindness plan which lists DR as a priority.

From 11th June to 14th June 2018, stakeholders met to draw guidelines for DR management at the various levels of the existing health system. These guidelines cover primary prevention, secondary prevention and tertiary prevention of visual impairment from DR. They also cover the treatment of DR, referral and periodic follow-ups. Guidelines for DR management are in the final stages of completion. National protocols for DR screening and treatment are currently in preparation and almost ready.

Ghana has a nationwide information management system, the District Health Information Management System (DHIMS) which now captures DR treatment services as of April 2018. It captures information on the number of retinal lasers done, and the number of vitreoretinal surgeries done.

Some DR treatment services covered by the National Health Insurance Scheme are diabetes care and DR screening. Optometrists can play the following roles in DR treatment services: Health Education; Awareness Creation; DR screening; DR Grading; DR Counselling.

## Discussion

This novel study presents the situation analysis of DR treatment services in Ghana and provides evidence on the breadth, coverage, workload and gaps in service delivery for DR treatment in Ghana. The results show geographical variation in the distribution of DR services, equipment, and human resource across Ghana. Furthermore, the price of DR treatment (anti-VEGF) was significantly influenced by the setting and regions in which patients are utilising these services.

The estimated burden of diabetes mellitus is 3.6% of the general population aged 20 years and above and for Ghana, which translates to 533,000 Ghanaians living with diabetes mellitus [18]. Of this number, an estimated 34% of them constituting about 181, 220 diabetics have DR [19] while about 53,300 about persons with diabetes representing 10% will have VTDR [19]. Considering that only 14 facilities offer DR treatment services at the end of December 2017, serving 53,330 people needing treatment for VTDR, will put a considerable burden on resources available in the various health facilities. Besides these numbers, the majority of the facilities are in Accra, the nation’s capital leaving other regions deprived of DR treatment services.

In Ghana, retinal lasers and intravitreal Anti-VEGF injections are given by Ophthalmologists and vitreoretinal surgeons, which is recommended by the ICO [20] although in other places like the United Kingdom, ophthalmic nurses are allowed to give Anti-VEGF injections [21, 22]. Our study indicates that VR surgeons and Ophthalmologists involved in DR treatment are not equally distributed around the country leaving gaps in DR treatment services between regions, urban and rural areas, which are consistent with findings from a similar study in the western province of Sri-Lanka in 2017 [8]. Of the 11 facilities with VR surgeons, 90.9% are located in the Greater Accra region and the remaining 9.1% in the Ashanti region leaving the rest of the country deprived of vitreoretinal services. DR patients in the northern part of the country will have challenges travelling down to health facilities in these regions located in the southern part of the country especially if they are from areas where resources are limited and road networks are bad. Reasons why VR surgeons are concentrated in these regions may be because these are the two most populous regions in the country. Together, these regions represent 34.87% of Ghana’s total population [23, 24].

Moreover, we found disparities in the distribution of the number of ophthalmologists that administer DR treatment services across the country. Our results are consistent with similar studies that reported huge service delivery gaps in developing countries [8, 25–27]. The World Health Organization (WHO) recommendation for human resource distribution for low-income countries, highlights that a minimum of four Ophthalmologists should serve a million population [26]. In Ghana, there are 91 ophthalmologists serving a population of approximately 30 million [28]. This number is less than what is needed. Furthermore some Ophthalmologists are not specialized in delivery of care for DR. Evidence from the current eye health systems assessment report [29] for Ghana showed an inequitable distribution of ophthalmologist across the country with many regions being understaffed. In addition, the report showed a limited number of sub-specialty (including experts in DR treatment) with the exception of paediatric ophthalmology [29]. Furthermore, a study analysing eye health delivery in Ghana by Morny and colleagues showed a positive skewness in the distribution of human resources across two regions (Ashanti and Greater Accra regions) and with limited specialist eye care (such as DR services) equipment across the nation [28]. Our findings show an under-utilisation of human resources, especially with regions like the Eastern, Central, Western, Upper East and Upper West having no access to any form of DR treatment services.

In terms of DR treatment facilities’ geographical location, most were concentrated in one region (Greater Accra Region), leaving the other regions underserved. Out of the total of ten administrative regions, five had no facilities providing DR services. Thus, the majority of patients have no access to services within their reach.

The lack of available services also means that DR and VTDR may only get noticed at advanced stages and even then, they will have to travel at greater cost to reach these referral centres where these services are provided. This has cost implications, and patients who cannot afford the cost of travelling to seek these services will have to be left to their fate. This goes against the fundamental principle of universal health coverage which aims to provide financial risk protection for persons seeking health care.

We observed that the price of Anti-VEGF per eye for DR treatment differs significantly across the facility’s geographical location and regions. Our findings contrast with results from other similar studies which showed an association between the price of treatment and uptake of DR services [30–32]. For example, in a qualitative hospital-based survey in Nigeria, Ibrahim et al. [30] reported cost of DR treatment, including Anti-VEGF, was a disenabling factor for assessing DR treatment services. In addition, a study by Fletcher et al. [32] in rural India identified direct and/or indirect cost as a barrier to the lower uptake of DR services. Furthermore, in a randomised control trial among people with diabetes mellitus, Lian et al. [31] demonstrated that those with co-payment of service had lower DR screening uptake compared with subjects that received care with no charges. Our study shows that despite the higher cost of treatment, private centres located in the main cities delivered the majority of DR treatment in the country. This differs from the inverse care law [33], which states that “*the availability of good medical care tends to vary inversely with the need for the population served*”. There is some evidence that shows that diabetes prevalence in Ghana is higher in urban centres as compared to rural areas [5, 34, 35]. This creates a higher demand in the cities, where the population are also likely to have a higher purchasing power, generating more demand for DR treatment services.

On the other hand, our results indicate that uptake of treatment services was not dependent on the price of treatment but may have been influenced by other factors like setting (facility location) [36]. This study observed that facilities with higher prices for laser treatment did more lasers than those with the lowest prices. The difference observed was that a large proportion (99%) of the lasers were done in urban centres rather than in rural settings. The purchasing power of residents of urban areas is higher than those in rural areas, which may have influenced DR treatment services’ uptake within these settings.

Despite the study presenting preliminary nationwide data on DR treatment services, it still has some limitations. Our findings may have been underestimated since, in some facilities, the ophthalmologists admitted that not all completed treatments were imputed in the records folder and that they did more lasers and anti-VEGF treatments than were recorded. This could lead to underestimating people treated for DR. Facilities, which started DR treatment in 2018 and a private facility that was temporarily closed down at the time of the study were excluded.

## Conclusions

It is imperative to put measures in place to deal with DR given the disparities in the distribution of human resources and facilities for managing DR in Ghana. This study will aid in planning services for DR, ensuring resources are fairly distributed and access to treatment services are readily available countrywide. Services for DR should be incorporated into mainstream diabetes care so that treatment services will be readily accessible to people living with VTDR. DR treatment services should be included in NHIS packages allocated for diabetes complications to ease the financial burden of people with VTDR.

## Supplementary Information

**Additional file 1.**

**Additional file 2.**

**Additional file 3.**

**Additional file 4.**

## Data Availability

The datasets used and/or analysed during the current study are available from the corresponding author on reasonable request.

## Abbreviations

Anti-VEGF: Anti–vascular endothelial growth factor therapy
CHAG: Christian Health Association of Ghana
DR: Diabetic retinopathy
NGO: Non-Governmental Organisation
NHIS: National Health Insurance Scheme
VR: Vitreoretinal
VTDR: Vision-threatening diabetic retinopathy

## References

[1] Cho NH, Shaw JE, Karuranga S, Huang Y, da Rocha Fernandes JD, Ohlrogge AW, Malanda B (2018). IDF diabetes atlas: global estimates of diabetes prevalence for 2017 and projections for 2045. Diabetes Res Clin Pract.

[2] Whiting DR, Guariguata L, Weil C, Shaw J (2011). IDF diabetes atlas: global estimates of the prevalence of diabetes for 2011 and 2030. Diabetes Res Clin Pract.

[3] Mbanya JC, Motala AA, Sobngwi E, Assah FK, Enoru ST (2010). Diabetes in sub-Saharan Africa. Lancet.

[4] Popkin BM (1998). The nutrition transition and its health implications in lower-income countries. Public Health Nutr.

[5] Asamoah-Boaheng M, Sarfo-Kantanka O, Tuffour AB, Eghan B, Mbanya JC (2019). Prevalence and risk factors for diabetes mellitus among adults in Ghana: a systematic review and meta-analysis. Int Health.

[6] Lee R, Wong TY, Sabanayagam C (2015). Epidemiology of diabetic retinopathy, diabetic macular edema and related vision loss. Eye Vis (Lond).

[7] Sharma A (2017). Emerging simplified retinal imaging. Dev Ophthalmol.

[8] Piyasena PN, Murthy GV (2017). A situation analysis of diabetic eye care service delivery in health care institutions of the Western Province of Sri Lanka. Ceylon Med J.

[9] Amoah AG, Owusu SK, Acheampong JW, Agyenim-Boateng K, Asare HR, Owusu AA, Mensah-Poku MF, Adamu FC, Amegashie RA, Saunders JT (2000). A national diabetes care and education programme: the Ghana model. Diabetes Res Clin Pract.

[10] Poore S, Foster A, Zondervan M, Blanchet K (2015). Planning and developing services for diabetic retinopathy in sub-Saharan Africa. Int J Health Policy Manag.

[11] Blanchet K, Patel D (2012). Applying principles of health system strengthening to eye care. Indian J Ophthalmol.

[12] Blanchet K, Gilbert C, de Savigny D (2014). Rethinking eye health systems to achieve universal coverage: the role of research. Br J Ophthalmol.

[13] GSS (2012). Ghana Census 2010 Summary report.

[14] UN. World population prospects the 2017 revision (2017). https://population.un.org/wpp/publications/files/wpp2017_keyfindings.pdf.

[15] GSS (2015). Annual Gross Domestic Product.

[16] Cooke E, Hague S, McKay A (2016). The Ghana poverty and inequality report.

[17] GLSS (2019). Ghana living standards survey 7; GLSS.

[18] IDF (2017). International Diabetes Federation: Diabetes Atlas.

[19] Yau JW, Rogers SL, Kawasaki R, Lamoureux EL, Kowalski JW, Bek T, Chen SJ, Dekker JM, Fletcher A, Grauslund J (2012). Global prevalence and major risk factors of diabetic retinopathy. Diabetes Care.

[20] Wong TY, Sun J, Kawasaki R, Ruamviboonsuk P, Gupta N, Lansingh VC, Maia M, Mathenge W, Moreker S, Muqit MMK, Resnikoff S, Verdaguer J, Zhao P, Ferris F, Aiello LP, Taylor HR (2018). Guidelines on diabetic eye care: the International Council of Ophthalmology Recommendations for screening, follow-up, referral, and treatment based on resource settings. Ophthalmology.

[21] Michelotti MM, Abugreen S, Kelly SP, Morarji J, Myerscough D, Boddie T, Haughton A, Nixon N, Mason B, Sioras E (2014). Transformational change: nurses substituting for ophthalmologists for intravitreal injections - a quality-improvement report. Clin Ophthalmol.

[22] DaCosta J, Hamilton R, Nago J, Mapani A, Kennedy E, Luckett T, Pavesio C, Flanagan D (2014). Implementation of a nurse-delivered intravitreal injection service. Eye (Lond).

[23] GSS (2020). Population by Regions.

[24] GSS (2020). Population by regions.

[25] Murthy GV, Gilbert CE, Shukla R, Vashist P, Shamanna BR (2016). Situational analysis of services for diabetes and diabetic retinopathy and evaluation of programs for the detection and treatment of diabetic retinopathy in India: methods for the India 11-city 9-state study. Indian J Endocrinol Metab.

[26] Graham R (2017). Facing the crisis in human resources for eye health in sub-Saharan Africa. Community Eye Health.

[27] Afake H (2016). Impact of NHIS on the uptake of cataract services in the upper east region of Ghana. LondonSschool of Hygiene and Tropical Medicine.

[28] Morny EKA, Boadi-Kusi SB, Ocansey S, Kyei S, Yeboah K, Mmaduagwu MA (2019). Assessing the Progress towards achieving "VISION 2020: the right to sight" initiative in Ghana. J Environ Public Health.

[29] GHS (2013). Eye health systems assessment (EHSA): Ghana country report.

[30] Ibrahim OA, Foster A, Oluleye TS (2015). Barriers to an effective diabetic retinopathy Service in Ibadan, Nigeria (sub -Saharan Africa) - a pilot qualitative study. Ann Ib Postgrad Med.

[31] Lian JX, McGhee SM, Gangwani RA, Hedley AJ, Lam CL, Yap MK, Lai WW, Chu DW, Wong DS (2013). Screening for diabetic retinopathy with or without a copayment in a randomized controlled trial: influence of the inverse care law. Ophthalmology.

[32] Fletcher AE, Donoghue M, Devavaram J, Thulasiraj RD, Scott S, Abdalla M, Shanmugham AK, Murugan PB (1999). Low uptake of eye services in rural India: a challenge for programs of blindness prevention. Arch Ophthalmol.

[33] Hart JT (1971). The inverse care law. Lancet.

[34] Gatimu SM, Milimo BW, Sebastian MS (2016). Prevalence and determinants of diabetes among older adults in Ghana. BMC Public Health.

[35] Kweku M, Nyavor P, Bani F, Kudzo Axame W, Owusu R, Takramah W, Takase M, Tarkang E, Adjuik M (2017). Prevalence and awareness of type 2 diabetes among urban and rural traders in Hohoe municipality, Ghana. Int J Clin Case.

[36] Melese M, Alemayehu W, Friedlander E, Courtright P (2004). Indirect costs associated with accessing eye care services as a barrier to service use in Ethiopia. Tropical Med Int Health.

