# Supplementary material for: Situational analysis of diabetic retinopathy treatment Services in Ghana
Source: BMC Health Serv Res. 2021 Jun 17;21:584. doi: 10.1186/s12913-021-06608-9 (PMC8212523; doi:10.1186/s12913-021-06608-9)
Supplement: Supplementary file 1 — Additional file 1. [file 12913_2021_6608_MOESM1_ESM.docx]

Questionnaire ID No:

CHECKLIST

[0] [1] [3]

No Yes Not Available for examination

1. screening protocols and guidelines for diabetes:
2. screening protocols and guidelines for DR:
3. grading protocols and guidelines for DR:
4. treatment protocols and guidelines for DR:
5. DR treatment registry/records:
6. Anti-VEGF drugs:
7. 27 needle and 1ml syringe:
8. operating microscope:
9. retinal laser machine:
10. vitrectomy machine:
11. fundus/retinal camera:
12. OCT machine:
13. pan retinal photocoagulation lens:
14. FFA/ICG angiography:
15. slit lamp biomicroscope:
16. direct ophthalmoscope:
17. indirect ophthalmoscope:
18. 90 D lens:
19. 78 D lens:
20. 20 D lens:
21. three-mirror contact lens

[0] [1] [2]

No Yes Not functioning

1. visual acuity chart:
2. BP apparatus:
3. comment:
