# Supplementary material for: Situational analysis of diabetic retinopathy treatment Services in Ghana
Source: BMC Health Serv Res. 2021 Jun 17;21:584. doi: 10.1186/s12913-021-06608-9 (PMC8212523; doi:10.1186/s12913-021-06608-9)
Supplement: Supplementary file 2 — Additional file 2. [file 12913_2021_6608_MOESM2_ESM.docx]

**QUESTIONNAIRE**

1. Questionnaire ID No: 2. Region: [0] Greater Accra [1] Ashanti

[2] Northern

[3] Brong Ahafo

[4] Volta

[5] Eastern

[6] Western

[7] Central

[8] Upper East

[9] Upper West

1. Facility Name:
2. Town/City: Accra [0]

Kumasi [1]

Tamale [2]

Drobo [3]

Kpando [4]

others [5] specify:

1. Setting: a. Rural [0] 6. Sex: a. M [0] 7. Age:

b. Urban [1] b. F [1]

1. Role/Position of participant at Health facility:
2. Contact Email (optional):
3. Facility Type:
   1. MOH (Teaching, military or university Hospital): [0]
   2. Ghana Health Service: [1]
   3. Christian Health Association of Ghana: [2]
   4. Quasi: [3]
   5. NGO: [4]

f: Private: [5]

1. Please what guidelines do you use in managing Diabetic Retinopathy in you facility?
   1. ICO guidelines: [0]
   2. Clinical expertise: [1]
   3. others: [2] specify:
2. Are people attending diabetic clinics routinely referred for eye examinations? No [0]

Yes [1]

1. Referral pathway:
   1. one way: [0]
   2. reciprocal: [1]
2. Do you treat DR using any of the following?
   1. Retinal laser: [0]
   2. Anti-VEGF (Avastin): [1]
   3. Vitreo-retinal surgery: [2]
   4. Other Anti-VEGF: [3] specify:
3. Which categories of health professionals treat DR in this facility and how many?

[0] [1] [0] [1]

P: No Yes T: No Yes

|  |  |
| --- | --- |
|  |  |
|  |  |
|  |  |
|  |  |
|  |  |
|  |  |

|  |  |
| --- | --- |
|  |  |
|  |  |
|  |  |
|  |  |
|  |  |
|  |  |

- 1. vitreo-retinal surgeon: permanent: P
  2. ophthalmologist: part-time: T
  3. medical officer:
  4. optometrist:
  5. ophthalmic nurse:
  6. general nurse:
  7. others: specify:

1. Who does laser photocoagulation at the facility?

[0] [1] [0] [1]

P: No Yes T: No Yes

|  |  |
| --- | --- |
|  |  |
|  |  |
|  |  |
|  |  |
|  |  |
|  |  |

|  |  |
| --- | --- |
|  |  |
|  |  |
|  |  |
|  |  |
|  |  |
|  |  |

- 1. vitreo-retinal surgeon: permanent: P
  2. ophthalmologist: part-time: T
  3. medical officer:
  4. optometrist:
  5. ophthalmic nurse:
  6. general nurse:
  7. others: specify:

1. Who gives Anti-VEGF in this facility?

[0] [1] [0] [1]

P: No Yes T: No Yes

|  |  |
| --- | --- |
|  |  |
|  |  |
|  |  |
|  |  |
|  |  |
|  |  |

|  |  |
| --- | --- |
|  |  |
|  |  |
|  |  |
|  |  |
|  |  |
|  |  |

- 1. vitreo-retinal surgeon: [0] permanent: P
  2. ophthalmologist: [1] part-time: T
  3. medical officer: [2]
  4. optometrist: [3]
  5. ophthalmic nurse: [4]
  6. general nurse: [5]

d. others: [6] specify:

1. What training does your health professional have to treat DR with laser?
   1. specialist training [0]
   2. as part of professional training: [1]
   3. on the job training? [2]
   4. others: [3] specify:
2. What training does your health professional have to treat DR with Anti-VEGF?
   1. specialist training: [0]
   2. as part of professional training: [1]
   3. on the job training: [2]
   4. others: [3] specify:
3. How is continuing medical education provided to health professionals treating DR?
   1. formal training by a regulated body: [0]
   2. regular informal updates: [1]
   3. workshops: [2]
   4. updates on guidelines: [3]
   5. none: [4]
4. No. of people treated for DR with laser in 2017?
5. No. of eyes treated for DR with laser in 2017?
6. No. of people treated for DR with Anti-VEGF in 2017?
7. No. of eyes treated for DR with Anti-VEGF in 2017?
8. No. of people with DR treated by vitreo-retinal surgery in 2017?
9. No. of eyes with DR treated by vitreo-retinal surgery in 2017?
10. Do you have a register to record and monitor DR treatment services? No [0]

Yes [1]

[0] [1]

1. If yes, what indicators are monitored? No Yes

|  |  |
| --- | --- |
|  |  |
|  |  |
|  |  |
|  |  |

- 1. type of treatment:
  2. which eye is treated:
  3. no. of times the eye(s) is treated:
  4. visual acuity before and after treatment session:
  5. others: specify:

1. Are your services covered by the national health insurance scheme? No [0]

Yes [1]

[0] [1]

| 30. If yes, which of these services does it cover? | No Yes |  |
| --- | --- | --- |
| a. diabetes care: |  |  |
| b. DR screening: |  |  |
| c. laser photocoagulation: |  |  |
| d. treatment with Anti-VEGF: |  |  |
| e. vitreo-retinal surgery: |  |  |
| f. others: |  | specify: |

1. On average, how many sessions of laser photocoagulation is recommended?

|  |  |
| --- | --- |
|  |  |
|  |  |
|  |  |
|  |  |
|  |  |

1. What is the price of a regular laser photocoagulation session per eye (s)?
2. On average, how many sessions of Anti-VEGF is recommended?
3. What is the price of an Anti-VEGF session per eye (s)?
4. What role do you think optometrists can play in DR treatment services?
