# Supplementary material for: Situational analysis of diabetic retinopathy treatment Services in Ghana
Source: BMC Health Serv Res. 2021 Jun 17;21:584. doi: 10.1186/s12913-021-06608-9 (PMC8212523; doi:10.1186/s12913-021-06608-9)
Supplement: Supplementary file 3 — Additional file 3. [file 12913_2021_6608_MOESM3_ESM.docx]

Situation Analysis of Diabetic Retinopathy Treatment Services in Ghana

Survey Questionnaire

1. Questionnaire ID/No.
2. Region:
3. Contact email:
4. Are the any health facilities (Government, CHAG, NGO, Private, Quasi) with laser for Diabetic Retinopathy treatment in your region? Yes No

5. If yes, please name them:

6. Are the any health facilities (Government, CHAG, NGO, Private, Quasi) with Anti-VEGF for Diabetic Retinopathy treatment in your Region? Yes No

7. If yes, please name them:

8. Are the any health facilities (Government, CHAG, NGO, Private, Quasi) with Vitreoretinal surgery for Diabetic Retinopathy treatment in your Region? Yes No

9. If yes, please name them:

10. Do you have a vitreoretinal Surgeon in your Region? Yes No

11. If yes, please name which facilities they are base in:
