# Supplementary material for: Situational analysis of diabetic retinopathy treatment Services in Ghana
Source: BMC Health Serv Res. 2021 Jun 17;21:584. doi: 10.1186/s12913-021-06608-9 (PMC8212523; doi:10.1186/s12913-021-06608-9)
Supplement: Supplementary file 4 — Additional file 4. [file 12913_2021_6608_MOESM4_ESM.docx]

**QUESTIONNAIRE**

1. Questionnaire No: 2. Region:

1. Facility Name:
2. Town/City:
3. Setting: a. Rural 6. Sex: 7. Age:

b. Urban

1. Role/Position of participant at Health facility:
2. Contact Email (optional):
3. Facility Type:
   1. MOH (Teaching, military or university Hospital):
   2. Ghana Health Service:
   3. Christian Health Association of Ghana:
   4. Quasi:
   5. NGO:
   6. Private:

h. Others: specify:

1. Do you have a national health plan?
2. Is diabetes listed as a priority in the national health plan? 13.Do you have a national diabetes plan?
3. If yes, what does the plan cover?
   1. primary prevention of diabetes:
   2. complications (including vision impairment)
   3. community awareness and patient education
   4. clinical care, services and supplies:
   5. Not sure:

No Ye Not sure

1. Is there a national prevention of blindness plan?
2. If yes, is diabetic retinopathy listed as a priority in the plan?
3. Are there guidelines for diabetic retinopathy management?
4. If yes, please describe them:

No Ye Not sure

1. If yes to Q 17, what does it cover?
   1. prevention of vision loss from diabetic retinopathy:
   2. treatment:
   3. referral:
   4. periodic follow-up:
2. What levels of the health care system are these guidelines applicable?
   1. primary:
   2. secondary:
   3. tertiary:
   4. not sure: specify:
3. Are there national protocols for DR screening and Treatment?
4. Is there a national information management system?
5. If yes to Q22, are DR services captured?
6. If yes to Q23, which of these indicators does it monitor?
   1. number of people treated with laser for DR
   2. number of eye treated with laser for DR
   3. number of people treated with Anti-vitreal injections for DR
   4. number of people treated for DR by VR surgery
   5. number of eyes treated for DR by VR surgery

No Yes Not sure

1. Briefly describe the networks between services for diabetes care, DR screening and DR treatment:
2. Are DR services covered by the national health insurance scheme?
3. If yes, which of these services does it cover?

No Ye Not sure

- 1. diabetes care:

|  |  |  |
| --- | --- | --- |
|  |  |  |
|  |  |  |
|  |  |  |
|  |  |  |

- 1. DR screening:
  2. laser photocoagulation:
  3. treatment with Anti-VEGF:
  4. vitreo-retinal surgery:

1. What role do you think optometrists can play in DR treatment services?
